# Creation of an Open-Access, Mutation-Defined Fibroblast Resource for Neurological Disease Research

**DOI:** 10.1371/journal.pone.0043099

**Published:** 2012-08-27

**Authors:** Selina Wray, Matthew Self, NINDS Parkinson's Disease iPSC Consortium, NINDS Huntington's Disease iPSC Consortium, NINDS ALS iPSC Consortium, Patrick A. Lewis, Jan-Willem Taanman, Natalie S. Ryan, Colin J. Mahoney, Yuying Liang, Michael J. Devine, Una-Marie Sheerin, Henry Houlden, Huw R. Morris, Daniel Healy, Jose-Felix Marti-Masso, Elisavet Preza, Suzanne Barker, Margaret Sutherland, Roderick A. Corriveau, Michael D'Andrea, Anthony H. V. Schapira, Ryan J. Uitti, Mark Guttman, Grzegorz Opala, Barbara Jasinska-Myga, Andreas Puschmann, Christer Nilsson, Alberto J. Espay, Jaroslaw Slawek, Ludwig Gutmann, Bradley F. Boeve, Kevin Boylan, A. Jon Stoessl, Owen A. Ross, Nicholas J. Maragakis, Jay Van Gerpen, Melissa Gerstenhaber, Katrina Gwinn, Ted M. Dawson, Ole Isacson, Karen S. Marder, Lorraine N. Clark, Serge E. Przedborski, Steven Finkbeiner, Jeffrey D. Rothstein, Zbigniew K. Wszolek, Martin N. Rossor, John Hardy

**Affiliations:** 1 Department of Molecular Neuroscience, University College London Institute of Neurology, London, United Kingdom; 2 Coriell Institute for Medical Research, Camden, New Jersey, United States of America; 3 Dementia Research Centre, Department of Neurodegenerative Diseases, University College London Institute of Neurology, London, United Kingdom; 4 Department of Clinical Neuroscience, University College London Institute of Neurology, London, United Kingdom; 5 Cardiff University School of Medicine, University of Cardiff, Cardiff, United Kingdom; 6 Hospital Donastia, San Sebastian, Spain; 7 National Institute for Neurological Disorders and Stroke, National Institutes of Health, Bethesda, Maryland, United States of America; 8 Departments of Neurology and Neuroscience, Mayo Clinic Jacksonville, Jacksonville, Florida, United States of America; 9 Department of Neurology, Center for Movement Disorders, Ontario, Canada; 10 Department of Neurology, Medical University of Silesia, Katowice, Poland; 11 Department of Geriatric Psychiatry, Lund University, Lund, Sweden; 12 Department of Neurology, University of Cincinnati, Cincinnati, Ohio, United States of America; 13 Department of Neurological and Psychiatric Nursing, Medical University of Gdansk, Gdansk, Poland; 14 Department of Neurology , West Virginia University, West Virginia, United States of America; 15 Department of Neurology, Mayo Clinic, Rochester, Minnesota, United States of America; 16 Division of Neurology, Pacific Parkinson's Research Centre, University of British Columbia, Vancouver, British Columbia, Canada; 17 Department of Neurology and Neuroscience, School of Medicine, Johns Hopkins University, Baltimore, Maryland, United States of America; 18 Department of Psychiatry and Behavioural Sciences, John Hopkins University School of Medicine, Baltimore, Maryland, United States of America; 19 Neuroregeneration Program, Institute of Cell Engineering, Department of Neurology and the Solomon H. Snyder Department of Neuroscience, John Hopkins University, Baltimore, Maryland, United States of America; 20 Center for Neuroregeneration Research, Harvard Medical School, Belmont, Massachusetts, United States of America; 21 Department of Neurology, Psychiatry, Sergievsky Center, and Taub Institute, College of Physicians and Surgeons, Columbia University, New York, New York, United States of America; 22 Center for Motor Neuron Biology and Diseases, Departments of Neurology, Pathology and Cell Biology, College of Physicians and Surgeons, Columbia University, New York, New York, United States of America; 23 Baylor College of Medicine, Department of Genetics, Houston, Texas, United States of America; 24 Gladstone Institute of Neurological Disease, Taube-Koret Center for Huntington's Disease Research, Departments of Neurology and Physiology, University of California San Francisco, San Francisco, California, United States of America; 25 For a full list of the members of the NINDS Parkinson's Disease iPSC Consortium, NINDS Huntington's Disease iPSC Consortium, and NINDS ALS iPSC Consortium please see the Acknowledgments section; University of South Florida, United States of America

## Abstract

Our understanding of the molecular mechanisms of many neurological disorders has been greatly enhanced by the discovery of mutations in genes linked to familial forms of these diseases. These have facilitated the generation of cell and animal models that can be used to understand the underlying molecular pathology. Recently, there has been a surge of interest in the use of patient-derived cells, due to the development of induced pluripotent stem cells and their subsequent differentiation into neurons and glia. Access to patient cell lines carrying the relevant mutations is a limiting factor for many centres wishing to pursue this research. We have therefore generated an open-access collection of fibroblast lines from patients carrying mutations linked to neurological disease. These cell lines have been deposited in the National Institute for Neurological Disorders and Stroke (NINDS) Repository at the Coriell Institute for Medical Research and can be requested by any research group for use in *in vitro* disease modelling. There are currently 71 mutation-defined cell lines available for request from a wide range of neurological disorders and this collection will be continually expanded. This represents a significant resource that will advance the use of patient cells as disease models by the scientific community.

## Introduction

Neurodegenerative diseases, including Alzheimer's disease (AD), Parkinson's disease (PD), frontotemporal dementia, amyotrophic lateral sclerosis (ALS), Huntington's disease (HD), ataxias and dystonias are a major socioeconomic problem, and understanding the biological basis of neuronal death in these disorders is a major challenge for basic research. Many of the loci responsible for early-onset, familial forms of these disorders have been identified. Mutations in *APP*, *PS1* and *PS2* are associated with AD [1]–[4], *SNCA*, *LRRK2*, *PRKN*, *PINK1* and *GBA* [5]–[9] are associated with PD; *SOD1*, *TARDP* and *FUS* mutations lead to familial ALS [10]–[12]; frontotemporal dementia and parkinsonism linked to chromosome-17 is associated with *MAPT* (FTDP-17T) and *PGRN* mutations (FTDP-17U/GRN) [13]–[15]; and CAG expansion of the *HTT* gene causes HD [16].

Using this genetic information as a basis for developing cell and animal models has greatly enhanced our understanding of the biological mechanisms underlying neuronal degeneration in these disorders. However, current cell models of neurological disease are limited by two major drawbacks: non-physiological protein expression levels and/or a non-neuronal cell type [17]–[19]. Patient-derived cells such as fibroblasts have been used as models in several studies looking at the basis of neurological disorders, including AD [20]. Recently, human somatic cells, such as fibroblasts, were reprogrammed to pluripotency by the exogenous expression of the transcription factors OCT4, SOX2, KLF4 NANOG, LIN28 and MYC [20]–[22]. These induced pluripotent stem cells (iPSC) can be subsequently differentiated into neurons and glia, therefore by generating iPSC from patients carrying disease-linked mutations physiological expression of mutated genes in the cell type specifically affected in disease can be achieved. This technology has already been used to successfully model a range of neurological diseases including AD, PD, ALS and Ataxia [23]–[27].

Despite the fact that many of these diseases are adult onset, several groups have used iPSCs to model aspects of disease pathology. Perhaps the most notable of these is AD, where cells derived from patients with mutations in several genes have successfully recapitulated common pathology. Neurons generated from patients carrying point mutations in PSEN1, APP duplications and trisomy 21 (and thus an extra copy of the APP gene) each faithfully recapitulate features of AD pathology including increased Aβ production and elevated tau phosphorylation [26], [28], [29]. The presence of overlapping phenotypes in multiple patients with the same mutation, as well as mutations in different genes linked to the same disease, provides increased confidence that iPSC can be used to reveal disease phenotypes. Importantly, gamma secretase inhibitors prevented increased Aβ production in these cells, demonstrating the suitability of iPSC-neurons as a platform for drug screening [26], [29].

Further, iPSC have provided evidence for the importance of correct cellular context in disease models. Spinocerebellar ataxia type 3 is caused by an expansion of a polyglutamine coding repeat in the *ATXN3* gene. iPSC-neurons generated from SCA3 patients recapitulate the pathological hallmark of SCA3 patients: accumulation of detergent-insoluble aggregates of full length and cleaved Ataxin 3 [25]. This phenotype was specific to neurons, and furthermore was dependent on the presence of functional ion channels, demonstrating the ability of iPSC to uncover disease mechanisms by allowing the study of mutations in the context of functional human neurons.

The use of iPSC as disease models is reviewed comprehensively by Cherry et al [30]. There is now compelling evidence of the power of patient-derived iPSC to model disease pathology, offer insight into disease mechanisms and act as a platform for drug screening. However, it has also become apparent that there is extensive intra- and inter- patient variability (23, 25), and it is necessary to use both multiple iPSC lines per patient and multiple patients per gene in order to reliably assign disease phenotypes.

Although the sporadic forms of AD, PD and ALS are common, the familial forms caused by defined mutations are relatively rare, and for many research groups interested in these and other rarer neurological diseases, the limiting factor in the use of iPSC is access to patient fibroblasts with the disease-causing mutations of interest. For HD, where all affected individuals have the same type of mutation, an expanded CAG trinucleotide repeat, it is desirable to have access to subjects with a range of expansion size, which is the primary determinant of the rate of pathogenesis. Furthermore, recent reports have demonstrated the necessity of using multiple patient lines with mutations in the same gene, in order to ensure that observed cellular phenotypes are caused by the genetic lesion of interest and not patient variability [25], [26]. With this in mind, our goal was to generate a resource of fibroblast cell lines with mutations that are linked to neurological disease. There are currently 67 mutation-defined fibroblast lines available to request from the Coriell repository, and more lines currently undergoing expansion and quality control. These include cell lines with multiple different mutations in each specific gene as well as cell lines from multiple patients carrying the same mutation. Further lines will be collected and deposited as patients are identified in clinics for participation in this study. This represents a significant resource that will encourage the use of patient-derived cell models in research by the wider scientific community.

## Methods

### Patient consent and protection of privacy

In this study, for all biopsy samples taken, the subsequent generation and distribution of human cell lines, and the deposition of these cell lines in the NINDS repository were agreed by the patients using consent forms and patient information sheets that were reviewed and approved by local research ethics committees. Each sample is pseudoanonymised in a systematic way upon leaving the clinic. There are minor physical risks associated with the skin punch biopsy procedure, including the possibility of infection. These risks, as well as the relative benefits of participating in this study are also discussed with participants during the informed consent process. It is stressed that immediate benefits to the patients themselves are unlikely, but use of these cell lines for *in vitro* research will lead to an overall enhancement of our understanding of the basic disease mechanisms. In the future, this could result in the development of novel therapeutics. For some lines, consent specifically includes commercial use of the cells and pathogenic pathway discovery (but not for direct cellular therapeutics). However, cell lines will not be sold for profit and patients are informed that they will not benefit financially from any products or tests that arise from the use of these cells. We have found that patients were typically enthusiastic about participation in this study, and we are confident that we will expand our collection of patient-derived cell lines in the future.

### Fibroblast generation

Fibroblasts were generated from a 3–6 mm skin punch biopsy taken under local anaesthetic following informed consent. Biopsies were dissected into ∼1 mm pieces and cultured in 5 cm^2^ petri dishes in DMEM, 10% FBS, 1% L-Glutamine until fibroblasts were seen to grow out from the explants. When fibroblasts reached confluency, they were detached from culture dishes using TrypleE (Invitrogen) and transferred to larger culture vessels for further expansion. Cells are frozen at the lowest passage possible while still obtaining an adequate number of total cells for distribution (typically 2–4 passages or approximately 2×10^7^ total cells; cells are distributed at 5×10^5^ cells per ampoule). The passage number of the cells on distribution depends on demand for a particular cell line, however 40–60 ampoules of cells are generally derived per biopsy, whilst keeping the passage number between 2–4. Cells will be distributed at the lowest available passage, which is indicated for each sample listed in the Repository online catalogue.

### Quality control of fibroblast cultures

Fibroblast cultures are tested for Mycoplasma contamination prior to frozen storage, and after recovery from liquid nitrogen prior to distribution. The gender of cell lines is verified by PCR with a Y chromosome-specific primer pair. Replicate cultures or matched cultures of differing cell types from the same individual are analyzed by PCR using microsatellite and Y chromosome-specific primer pairs to assure cell culture identity.

### Immunocytochemistry

Fibroblasts were fixed in 4% paraformaldehyde for 30 min at room temperature then blocked and permeabilised in blocking buffer (10% FBS, 0.1% Triton X-100 in phosphate buffered saline) for 30 min at room temperature. Cells were incubated with rabbit polyclonal anti-FSP1 (1∶100, Abcam) and mouse monoclonal anti-human fibroblasts clone TE-7 (1∶100, Millipore) diluted in blocking buffer overnight at 4°C. Cells were then incubated with Alexa Fluor 488 and 568 antibodies (1∶500) for 1 h at room temperature and nuclei were stained using DAPI. Images were acquired using a Zeiss LSM 710 confocal microscope.

### Western blotting

Cells were washed in PBS and then lysed on ice for 30 minutes in lysis buffer (50 mM Tris-HCl, 150 mM NaCl, 1% v/v Tween-20, 0.2% NP40, 10%v/v Glycerol) containing Complete protease inhibitor cocktail (Roche). Lysates were centrifuged for 10 min at 11,000 g_(av)_, 4°C and protein concentrations were estimated using the BioRad DC Protein Assay Kit. Equal amounts of protein were electrophoresed on NuPAGE 4–12% Bis-Tris Gels (Invitrogen) and transferred onto nitrocellulose membranes (Whatman). Membranes were probed with primary antibodies to FSP-1 (rabbit polyclonal, 1∶500, Abcam) and β-actin (mouse monoclonal, 1∶5000, Sigma Aldrich) overnight at 4°C. Membranes were then incubated with appropriate secondary antibodies (AlexaFluor 680 anti-mouse IgG, Invitrogen and IRDye 800 anti-rabbit IgG, Rockland Immunochemicals, both 1∶5000) for 1 h at RT before visualisation using an Odyssey Infrared imaging system (LI-COR Biosciences).

### Population doubling levels

Population doubling level (PDL) is a measurement of the total number of times the cells within the population have doubled since their primary isolation. PDLs were calculated using the following equation:
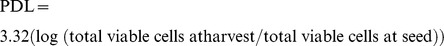
The total viable cells at seed was determined at the first seeding following proliferation of cells from the skin explant, or from the frozen ampoule for fibroblast cultures generated outside of Coriell. The total number of viable cells at harvest was determined immediately prior to cryopreservation.

## Results

### Collection of fibroblast cell lines

We have generated a collection of fibroblast cell lines from patients with mutations that are linked to neurodegenerative disorders, including AD, PD, ALS, FTD, HD, dystonias and ataxias. Also included in the collection are idiopathic sporadic Parkinson's disease fibroblast lines and normal control fibroblast lines, including family members of mutation carriers. These have been deposited in the National Institute for Neurological Disorders and Stroke (NINDS) Repository at the Coriell Institute for Medical Research (Camden, NJ) and the lines carrying known mutations are detailed in Table 1. Access to these cell lines is open to the scientific community and they are available to all researchers for use in basic research. This collection will be continually expanded and will be a valuable resource for research into basic disease mechanisms of neurological disorders. An up to date list of lines available upon request from the NINDS Repository can be found at: http://ccr.coriell.org/sections/collections/NINDS/FibroSubcollList.aspx?SsId=10&PgId=681.

**Table 1:** Fibroblast lines generated in this study.

Clinical	Gene	Inheritance	Mutation	Number of lines	Status	Reference
AD	*PSEN1*	D	Y115H	1	Submitted	[33]
	*PSEN1*	D	M146I	1	Available	[34]
	*PSEN1*	D	E184D	1	Available	[35]
	*PSEN1*	D	P264L	1	Available	[33]
	*PSEN1*	D	R278I	1	Submitted	[36]
PD	*SNCA*	D	Triplication	1	Available	[37]
	*LRRK2*	D	R1441G	2	Available	
	*LRRK2*	D	R1441C	2	Available	[38], [39]
	*LRRK2*	D	G2019S	20	19 Available	[40]–[42]
	*LRRK2*	D	G2019S homozygote	2		[43]
	*GBA*		N370S	4	Available	[42], [44]
	*GBA*		L444P	1		[42], [44]
	*PARK2*	R	R42P, ΔExon 3	1	Available	[45], [46]
	*PARK2*	R	Δ255, ΔExon 3–4	1	Available	[45], [46]
	*PARK2*	R	ΔExon 3–4 homozygote	1	Submitted	
	*PARK2*	R	R275W/R275Q	1	Available	[47]
	*PINK1*	R	Q456X homozygote	1		
	*PINK1*	R	D525N/W577R	1		
HD	*HTT*	D	CAG repeat, range 38–57	17	Available	
ALS	*SOD1*	D	A4V	2		
	*SOD1*	D	C38G	1		
	*SOD1*	D	L38V	1	Available	
	*SOD1*	D	E49K	1		
	*SOD1*	D	G86R	1		
	*SOD1*	D	A89V	1		
	*SOD1*	R	D90A	2	Available	
	*SOD1*	D	D91A	1	Available	
	*SOD1*	D	E100G	1	Available	
	*SOD1*	D	N138K	1		
	*SOD1*	D	I112T	1		
	*SOD1*	D	I113T	6	2 Available	
	*SOD1*	D	L144P	2	1 Available	
	*SOD1*	D	V148G	1	Available	
	*TARDBP*	D	G298S	1	Available	
FTDP-17T	*MAPT*	D	P301L	2	Available	[48]
	*MAPT*	D	V337M	2	Available	[49]
	*MAPT*	D	N279K	1	Available	[50], [51]
	*MAPT*	D	Exon 10+16	5		[52]
	*MAPT*	D	R406W	2		[53]
FTDP-17U	*GRN*	D	A9D	1	Available	[54]
	*GRN*	D	R493X	1		[55]
FTD	*VCP*	D	R155H	1		[56]
Perry Syndrome	*DCTN1*	D	T72P	1	Available	[57], [58]
Dystonia	*THAP1*	D	I149T	1		[59], [60]
Ataxia	*CACNA1A*	D	R1346X	1		

Disease, gene, mutation and mode of inheritance for fibroblast cell lines. The current status of each line (available, submitted but not yet in catalogue) is indicated. Where the status is left blank, this indicates fibroblast lines have been generated but are awaiting submission to the NINDS repository. All variants are heterozygous unless otherwise stated. References indicate where families have been described in the literature. D = autosomal dominant, R = autosomal recessive.

Fibroblast cell lines are deposited along with a clinical data elements (CDE) form that outlines the clinical background of the patient from whom the cells are derived. This protects the identity of the patient (see below) while providing the end-user with confidence in the clinical diagnosis. CDE's for PD, ALS, and HD have been developed with input from researchers in the field. For AD and other dementia cell lines, there is currently no CDE; however, information (e.g., sex, year of birth, and MMSE score at the time of biopsy) is included.

Fibroblast cultures are available upon request to all research laboratories, including those in industry. Users wishing to request cells are asked to complete a statement of research intent and complete a NINDS Repository Materials Transfer Agreement (MTA).

### Skin explant-derived cell lines express the fibroblast-specific proteins FSP1 and TE7

**Figure 1:**
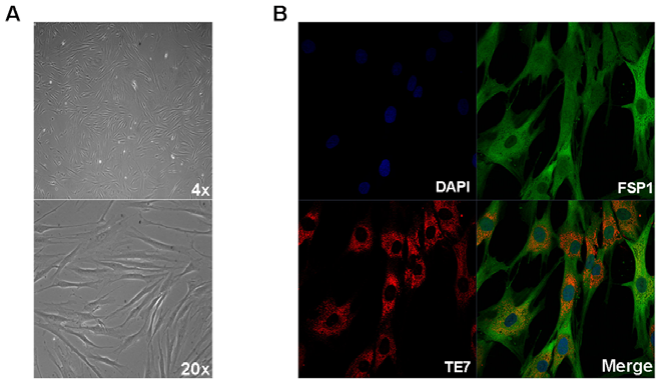
Fibroblast cultures express the mesenchymal markers FSP1 and TE7. Cells generated from skin punch biopsies were verified as fibroblasts by morphological assessment (A) and positive staining with antibodies to fibroblast-specific protein 1 (FSP1) and fibroblast-specific clone TE7 (B, 63×). All fibroblasts examined (n = 6) demonstrated positive staining with both antibodies.

**Figure 2:**
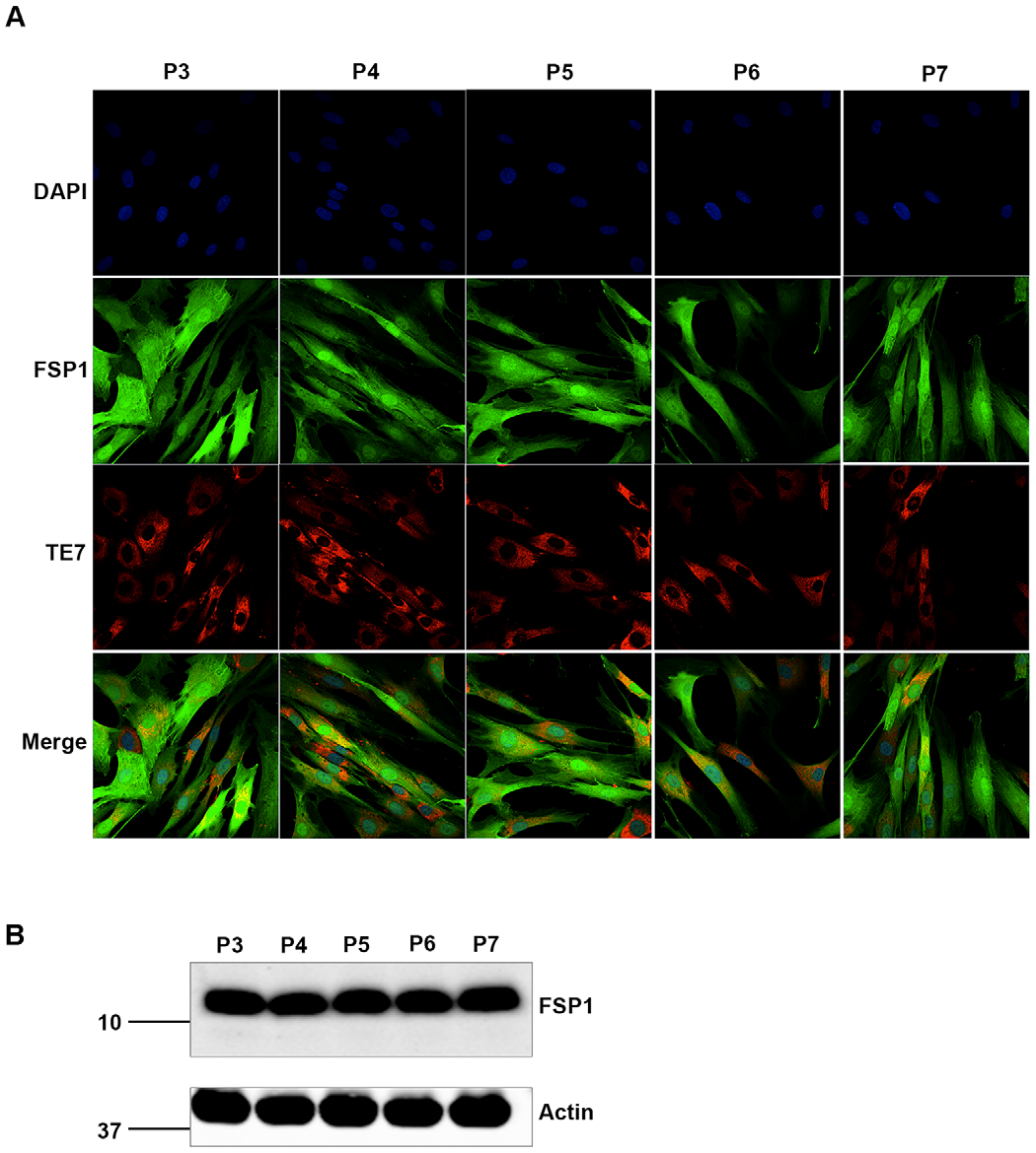
Fibroblast morphology and marker expression remain consistent during prolonged culture. Fibroblast lines were immunostained with antibodies FSP1 and TE7 at multiple consecutive passages (A). Passage numbers are indicated above the panels. Morphology, FSP1 and TE7 staining did not change during five consecutive subculturings (n = 6, representative images from line NM34737, carrying the PSEN1 M146I mutation are shown). FSP1 levels were also detected by western blotting of fibroblast cell lysates (B). FSP1 was detected as a single band at 12 kDa in all fibroblast lines examined (top panel, n = 6). β-actin was used as a loading control (bottom panel). No variation in FSP1 levels was observed between passages or between cell lines.

For all fibroblast lines generated, the identity and purity of each line was confirmed by assessment of characteristic spindle-shaped morphology (Fig. 1A) [31]. We also immunostained a subset of lines (n = 6) for fibroblast-specific protein 1 (FSP1) and TE-7, which detects an epitope specifically expressed by cells that are mesenchymal in origin. All fibroblast lines examined showed strong cytoplasmic staining of both FSP1 and TE7, confirming that cells cultures established from skin explants are indeed fibroblasts (Fig. 1B). Next, we examined the expression of FSP1 and TE-7 over multiple passages, to ensure that the properties of the fibroblast lines were not altered by increased time in culture. We found that the morphology of fibroblast lines remained unchanged throughout five consecutive passages. Likewise, FSP1 and TE-7 were highly expressed in all cells and did not show altered levels, or altered distribution, during continuous culture (Fig. 2A). FSP1 levels were also examined by western blot (Figure 2B). In fibroblast cell lysates, FSP1 was detected as a single band at the expected molecular weight of 12 kDa (Fig. 2B). FSP1 was expressed at high levels in all cell lines examined (n = 6) and the levels of FSP1 were not different between cell lines, or between different passages.

### Population doubling levels

**Figure 3:**
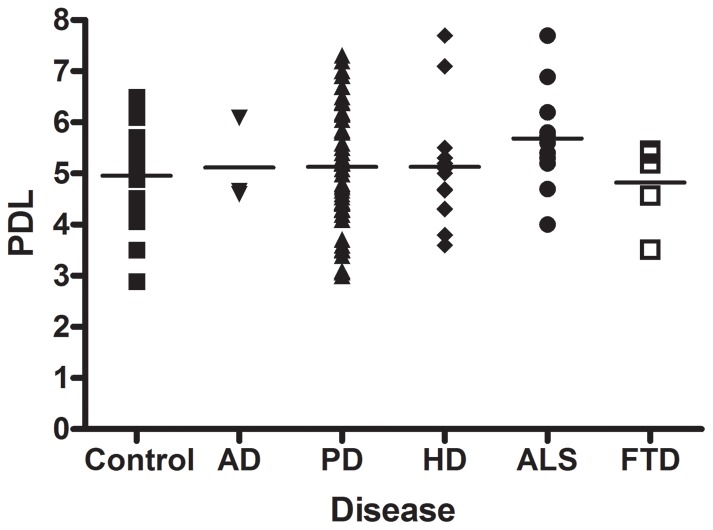
Population doubling levels of fibroblast cell lines. Population doubling levels were calculated for each of the cell lines available in the NINDS repository at the time of cryopreservation. Individual points of the graph correspond to the PDL of individual fibroblast lines, the horizontal line represents the mean PDL for each disease category. PDLs ranged between 2–8 with a mean PDL of ∼5 for both control and disease cell lines. A full list of PDLs for individual cell lines in provided in Table S1.

Fibroblasts have a limited proliferative lifespan in culture, and are able to complete a finite number of cell divisions before reaching senescence (the Hayflick limit) [61]. As passage number is a reflection only of the number of times a particular cell line has been subcultured, and not a reflection of the absolute time in culture of that particular cell line, the population doubling level (PDL) of each fibroblast line available in the NINDS catalogue was determined. PDL is a measure of the total number of times a cell population has doubled since its initial isolation *in vitro*. The PDLs of fibroblast lines in our collection varied from 2.89–7.7 (Table S1). Fig. 3 shows the range and mean PDLs of the control fibroblasts, and fibroblast lines from each disease group. A similar range of PDL variability was seen across all disease groups and the mean PDLs of fibroblasts were ∼5 for each of the categories represented by the collection. Thus, fibroblast lines requested from the NINDS repository are comparable in terms of the absolute time in culture of the cell. Senescence of human diploid fibroblast cultures does not occur until after 40–50 population doublings [62]. Therefore, cell cultures within our collection have low population doubling numbers and can be expanded sufficiently by the end-users prior to senescence. Furthermore, although the proliferative capacity of the starting cell population may impact on reprogramming efficiency, both our control and disease lines should retain sufficient proliferative capacity to be suitable for reprogramming to iPSCs.

## Discussion

The search for the genetic basis of disease has provided the impetus for the generation of animal and cell models that recapitulate key disease features and allow better understanding of the underlying biological mechanisms leading to cell death. A major challenge to understanding the basis of neurological disorders is our ability to model disease causing mutations at physiological levels, in a relevant cell type. The recent development of iPSCs, which can subsequently be differentiated into neurons and glial cells, is redefining the way we approach *in vitro* modelling of neurological disorders. We have developed a collection of primary fibroblast lines from patients carrying mutations that are associated with neurological disorders that can be accessed by all bona fide research groups.

Although others have developed collections of disease-specific iPSCs [32], we focussed on developing fibroblast cell lines. The cell lines in our collection express high levels of the fibroblast markers FSP-1 and TE-7, and are cryopreserved at low population doubling levels for distribution. However, although fibroblasts are the most common cell type in cultures established from dermal outgrowths, these cultures actually represent a heterogeneous cell population including endothelial cells, pericytes and several types of stem/progenitor cells [62]. This cellular diversity could influence the ability of each individual fibroblast line to give rise to iPSC.

The molecular mechanisms underlying the reprogramming of fibroblasts to iPSC are poorly understood and there has been much debate as to whether the process is stochastic (all cells within a given population have the potential to be reprogrammed) or elite (only a subset of cells with particular properties can be reprogrammed). In a recent study, Wakeo and colleagues determined that iPSC were exclusively generated from a sub-population of cells positive for both the stem cell marker SSEA3 and the mesencyhmal marker CD105 [63]. These cells, termed muse cells (multilineage-differentiating stress enduring cells), express the pluripotency markers Oct3/4, Nanog and Sox2 and represent approximately 2% of cells present in fibroblast cultures.

This work provides support for the elite model of reprogramming and suggests the efficiency of reprogramming from each of the fibroblast cultures within this collection may depend on the proportion of Muse cells present, which was not examined in this study. However, even in a pure Muse cell population the efficiency of reprogramming remains low (0.03%), and it therefore seems likely that there is some stochastic influence on reprogramming. This notion is supported by multiple reports describing the addition of extra reprogramming factors and small molecules that increase the efficiency of reprogramming (reviewed in [64]). Thus, the elite vs stochastic debate remains open, but it is important for research groups requesting cells described in this manuscript to be aware of the implications of fibroblast culture diversity. By making fibroblast lines available, the end-users retain the flexibility to reprogram by their method of choice.

This collection contains cell lines with mutations in a wide range of genes as well as multiple different mutations in each gene. In many cases, cell lines from several patients with the same mutation are available which will control for patient variability and allow robust phenotypes to be defined. The rarity of familial forms of neurological diseases means this represents a valuable resource which we anticipate will be widely used by the scientific community, advancing the use of patient cells for *in vitro* disease modelling.

## Supporting Information

Table S1 **Population doubling levels for fibroblast lines in the NINDS repository.** NINDS reference number, disease, mutation and population doubling level for each cell line currently available from the NINDS repository.
(DOCX)
