# Supplementary material for: Creation of an Open-Access, Mutation-Defined Fibroblast Resource for Neurological Disease Research
Source: PLoS One. 2012 Aug 27;7(8):e43099. doi: 10.1371/journal.pone.0043099 (PMC3428297; doi:10.1371/journal.pone.0043099)
Supplement: Table S1 — Population doubling levels for fibroblast lines in the NINDS repository. NINDS reference number, disease, mutation and population doubling level for each cell line currently available from the NINDS repository. (DOCX) [file pone.0043099.s001.docx]

**Table S1: Population doubling levels for fibroblast lines in the NINDS repository**

| Catalogue | Disease | Gene | Mutation | PDL |
| --- | --- | --- | --- | --- |
| ND29178 | Control |  |  | 6.1 |
| ND29179 | Control |  |  | 6.21 |
| ND29194 | Control |  |  | 3.5 |
| ND29510 | Control |  |  | 4.1 |
| ND29971 | Control |  |  | 6.5 |
| ND30014 | Control |  |  | 5.1 |
| ND30625 | Control |  |  | 4.4 |
| ND31008 | Control |  |  | 4.1 |
| ND31037 | Control |  |  | 5.7 |
| ND31845 | Control |  |  | 4.3 |
| ND32603 | Control |  |  | 6.3 |
| ND33391 | Control |  |  | 5.3 |
| ND34769 | Control |  |  | 5.43 |
| ND34770 | Control |  |  | 5.71 |
| ND34791 | Control |  |  | 4.06 |
| ND35044 | Control |  |  | 4.52 |
| ND34730 | AD | PSEN1 | E184D | 6.09 |
| ND34732 | AD | PSEN1 | M146I | 4.66 |
| ND34733 | AD | PSEN1 | P264L | 4.6 |
| ND27760 | PD | SNCA | Triplication | 4.45 |
| ND29370 | PD | LRRK2 | G2019S | 5.5 |
| ND29423 | PD | LRRK2 | G2019S | 7 |
| ND29492 | PD | LRRK2 | G2019S | 6.4 |
| ND29542 | PD | LRRK2 | G2019S | 3.07 |
| ND29802 | PD | LRRK2 | G2019S | 5.4 |
| ND31960 | PD | LRRK2 | G2019S | 5.3 |
| ND32949 | PD | LRRK2 | G2019S | 5.22 |
| ND32954 | PD | LRRK2 | G2019S | 4.54 |
| ND32970 | PD | LRRK2 | G2019S | 6.91 |
| ND32973 | PD | LRRK2 | G2019S | 4.99 |
| ND32975 | PD | LRRK2 | R1441G | 4.29 |
| ND32976 | PD | LRRK2 | R1441G | 5.8 |
| ND33879 | PD | LRRK2 | G2019S | 4.76 |
| ND33965 | PD | LRRK2 | G2019S | 4.41 |
| ND33995 | PD | LRRK2 | G2019S | 6.05 |
| ND34198 | PD | LRRK2 | G2019S | 6.2 |
| ND34235 | PD | LRRK2 | G2019S | 7.31 |
| ND34267 | PD | LRRK2 | G2019S | 6.16 |
| ND34810 | PD | LRRK2 | G2019S | 5.21 |
| ND34980 | PD | LRRK2 | G2019S | 4.43 |
| ND29756 | PD | GBA | N370S | 5.3 |
| ND31630 | PD | GBA | N370S | 4.6 |
| ND34263 | PD | GBA | N370S | 5.2 |
| ND34982 | PD | GBA | N370S | 5.84 |
| ND29369 | PD | PARK2 | R275W | 4.2 |
| ND29543 | PD | PARK2 | EX3-4DEL | 4.8 |
| ND29968 | PD | PARK2 | Q34R, R275W | 6.7 |
| ND29969 | PD | PARK2 | R275W | 5.85 |
| ND31618 | PD | PARK2 | R42P | 4.1 |
| ND35200 | PD | PARK2 | EX3DEL | 5.27 |
| ND29494 | iPD |  |  | 4.3 |
| ND29541 | iPD |  |  | 6.4 |
| ND30116 | iPD |  |  | 6.4 |
| ND30159 | iPD |  |  | 3.1 |
| ND31508 | iPD |  |  | 3 |
| ND31717 | iPD |  |  | 3.5 |
| ND32157 | iPD |  |  | 4.1 |
| ND32462 | iPD |  |  | 6.5 |
| ND32697 | iPD |  |  | 3.6 |
| ND33424 | iPD |  |  | 4.73 |
| ND33847 | iPD |  |  | 3.71 |
| ND34265 | iPD |  |  | 5.59 |
| ND34854 | iPD |  |  | 3.52 |
| ND35425 | iPD |  |  | 7.2 |
| ND35843 | iPD |  |  | 6.38 |
| ND35976 | iPD |  |  | 4.67 |
| ND37132 | iPD |  |  | 6.17 |
| ND37180 | iPD |  |  | 3.39 |
| ND29801 | HD | HTT | CAG:40 | 7.7 |
| ND29970 | HD | HTT | CAG:40 | 4.3 |
| ND30013 | HD | HTT | CAG:43 | 5.2 |
| ND30015 | HD | HTT | CAG:41 | 4.31 |
| ND30016 | HD | HTT | CAG:41 | 7.1 |
| ND30047 | HD | HTT | CAG41 | 5.1 |
| ND30259 | HD | HTT | CAG:38 | 5 |
| ND30260 | HD | HTT | CAG:38 | 5.5 |
| ND30422 | HD | HTT | CAG:40 | 5.3 |
| ND30626 | HD | HTT | CAG:41 | 5.1 |
| ND30967 | HD | HTT | CAG:43 | 5 |
| ND31009 | HD | HTT | CAG: 40,49 | 3.8 |
| ND31038 | HD | HTT | CAG:44 | 5.5 |
| ND31551 | HD | HTT | CAG:38 | 5.3 |
| ND31846 | HD | HTT | CAG:40 | 3.6 |
| ND33392 | HD | HTT | CAG:57 | 4.67 |
| ND33947 | HD | HTT | CAG:40 | 4.69 |
| ND29801 | HD | HTT | CAG:40 | 7.7 |
| ND29970 | HD | HTT | CAG:40 | 4.3 |
| ND29149 | ALS | SOD1 | D90A | 5.4 |
| ND29415 | ALS | SOD1 | L144P | 5.3 |
| ND29422 | ALS | SOD1 | I113T | 4 |
| ND29509 | ALS | SOD1 | D91A | 4.7 |
| ND29523 | ALS | SOD1 | L38V | 5.74 |
| ND29688 | ALS | SOD1 | E100G | 7.7 |
| ND29774 | ALS | SOD1 | D90A | 6.2 |
| ND32969 | ALS | SOD1 | I113T | 5.2 |
| ND30327 | ALS | FUS | R521G | 5.6 |
| ND32947 | ALS | TARDBP | G289S | 6.89 |
| ND32945 | FTD | MAPT | N279K | 4.57 |
| ND32951 | FTD | MAPT | V337M | 5.45 |
| ND32953 | FTD | PGRN | A9D | 5.39 |
| ND32955 | FTD | MAPT | P301L | 3.51 |
| ND32956 | FTD | MAPT | P301L | 5.2 |
